# MDAR: A Multiscale Features-Based Network for Remotely Measuring Human Heart Rate Utilizing Dual-Branch Architecture and Alternating Frame Shifts in Facial Videos

**DOI:** 10.3390/s24216791

**Published:** 2024-10-22

**Authors:** Linhua Zhang, Jinchang Ren, Shuang Zhao, Peng Wu

**Affiliations:** 1 Department of Computer Engineering, Taiyuan Institute of Technology, Taiyuan 030008, China; zhanglh@tit.edu.cn; 2 School of Computer Science and Technology, Taiyuan Normal University, Jinzhong 030619, China; m18403587717@163.com; 3 School of Computing, Engineering and Technology, Robert Gordon University, Aberdeen AB10 7QB, UK; jinchang.ren@ieee.org; 4 School of Computer and Information Technology, Beijing Jiaotong University, Beijing 100044, China

**Keywords:** rPPG, multiscale features, dual-branch architecture, alternating frame shifts

## Abstract

Remote photoplethysmography (rPPG) refers to a non-contact technique that measures heart rate through analyzing the subtle signal changes of facial blood flow captured by video sensors. It is widely used in contactless medical monitoring, remote health management, and activity monitoring, providing a more convenient and non-invasive way to monitor heart health. However, factors such as ambient light variations, facial movements, and differences in light absorption and reflection pose challenges to deep learning-based methods. To solve these difficulties, we put forward a measurement network of heart rate based on multiscale features. In this study, we designed and implemented a dual-branch signal processing framework that combines static and dynamic features, proposing a novel and efficient method for feature fusion, enhancing the robustness and reliability of the signal. Furthermore, we proposed an alternate time-shift module to enhance the model’s temporal depth. To integrate the features extracted at different scales, we utilized a multiscale feature fusion method, enabling the model to accurately capture subtle changes in blood flow. We conducted cross-validation on three public datasets: UBFC-rPPG, PURE, and MMPD. The results demonstrate that MDAR not only ensures fast inference speed but also significantly improves performance. The two main indicators, MAE and MAPE, achieved improvements of at least 30.6% and 30.2%, respectively, surpassing state-of-the-art methods. These conclusions highlight the potential advantages of MDAR for practical applications.

## 1. Introduction

As quality of life continues to improve, individuals are placing greater emphasis on their own and their family’s health [1]. Heart rate, a fundamental indicator for health monitoring and daily care, has garnered increasing attention. Heart rate, defined as the number of heartbeats per minute, reflects the basic state of an individual’s health. Traditional methods for measuring heart rate include bioelectrical potential-based measurements [2], photoplethysmography (PPG) [3], and vibration-based detection [4]. These methods have been widely adopted, particularly with the popularization of smartwatches and fitness trackers, making heart rate monitoring more accessible and convenient.

Although contact-based devices provide high accuracy, they require direct contact with the subject, which constrains their use in specific scenarios. Remote photoplethysmography (rPPG) [5] effectively addresses this limitation. As a non-contact heart rate monitoring technology, it uses video sensors to convert light signals, which reflect subtle color changes on the skin surface into electrical signals known as rPPG signals. Neural networks can analyze these signals to estimate blood flow, thereby predicting heart rate. This technology eliminates the need for physical contact, reduces the risk of cross-infection, and allows for the monitoring of physiological indicators in multiple individuals without drawing attention. It is particularly beneficial in crowded public spaces and security applications. Another typical application of this technology is in remote monitoring within isolated wards, supporting long-term, non-intrusive, continuous monitoring, which significantly reduces both time and financial costs. The rPPG technology has broad applications across various fields, including remote health monitoring [6], neonatal health monitoring [7], contactless biometric identification [8], activity monitoring [9], and security surveillance [10].

To further advance rPPG technology, many researchers have explored its underlying theories and practical applications, achieving notable progress. The concept of rPPG was first proposed in [5], demonstrating the feasibility of using regular cameras to remotely measure human heart rate. This research performed spectral analysis on the average pixel values of the RGB channels, identifying a significant frequency component in the 0.8–4.0 Hz range, corresponding to the human heart rate. In 2008, Verkruysse et al. [5] enhanced the rPPG signal using color space conversion and introduced methods for heart rate detection under natural or everyday indoor lighting conditions, avoiding the need for additional equipment. The study in [11] proposed an rPPG pulse rate estimation method based on chromatic information, achieving robust pulse rate detection in complex environments through linear transformation in the chromatic space and time-frequency analysis. The study in [12] used regular webcams and improved rPPG signal processing algorithms to enable the contactless measurement of multiple physiological parameters, such as heart and respiratory rates, thus expanding rPPG’s application in physiological health monitoring. The study in [13] improved the robustness and measurement accuracy of rPPG under movement conditions by analyzing the periodicity and BVP signal.

Despite the progress made in early rPPG methods, their performance and applicability remain limited by outdated architectures. Traditional methods often rely on manually designed signal-processing pipelines, such as filtering [14] and ICA separation [15], resulting in limited robustness. These methods typically assume that variations in facial blood flow are the most prominent periodic signals in a video. However, in complex environments, pixel variations caused by motion and lighting often violate this assumption, significantly reducing the accuracy of heart rate extraction. Furthermore, early methods generally considered only the RGB channel information and failed to fully exploit the spatiotemporal features present in facial videos. Traditional methods are often evaluated on small samples and specific scenarios, making it difficult to ensure generalization performance.

Faced with the limitations of traditional rPPG methods, deep learning technology has gradually become a breakthrough direction in this field. Unlike traditional methods, deep learning architectures can automatically learn features from data without human intervention, which greatly improves the flexibility and generalization of models. Standard deep learning architectures, such as convolutional neural networks (CNNs) [16] and recurrent neural networks (RNNs) [17], have been widely used for many computer vision tasks [18,19,20,21,22]. In the field of rPPG, deep learning not only reduces the dependence on complex preprocessing through automatic feature extraction, but also better captures spatiotemporal features in video and improves adaptability to environmental changes.

DeepPhys based on convolutional attention networks [23] was proposed. This method employs deep learning techniques to automatically learn salient features from facial videos, thereby eliminating the need for traditional manual feature engineering. The study in [24] developed two neural models for camera-based physiological measurements, which eliminated the need for complex preprocessing and demonstrated accuracy on three public datasets. However, these models still underperformed in low-light and motion-intensive environments. The study in [25] proposed an end-to-end method called PhysFormer, validated using four benchmark datasets. Compared to previous methods, PhysFormer exhibited better temporal dependency modeling capabilities; however, its computational complexity was high, requiring further optimization for real-time performance and robustness to occlusions and pose variations. In [26], a spatiotemporal network-based method for the remote measurement of photoplethysmography (PPG) signals from facial videos was proposed, which enhances the accuracy and robustness of physiological signal extraction by combining spatial and temporal information. The study in [27] introduced a multitask convolutional attention network, demonstrating high efficiency on two public datasets; however, it primarily focused on applications within the same device and showed limited robustness to ambient light changes. The study in [28] introduced a dual-branch measurement model that achieved good results on three datasets by utilizing high- and low-resolution branches. Nevertheless, further improvements are necessary to model the correlations between different tasks and to explore the model design space.

Although deep learning-based methods have addressed some limitations of traditional approaches and advanced rPPG technology, several challenges remain. First, facial blood flow changes caused by heartbeats are subtle and easily disrupted by environmental lighting variations and facial motions [29]. Second, the absorption and reflection characteristics of the skin vary significantly across different wavelengths, making it challenging to accurately extract heart rate-related blood flow signals from videos [30]. Additionally, physiological differences among individuals of different races and skin colors pose challenges to the robustness of rPPG methods. To further improve the performance of rPPG in complex scenarios, this study puts forwards a more efficient heart rate network aimed at addressing key issues such as ambient light variation and individual skin color differences, thereby providing a more robust remote measurement of heart rate. The main innovations of this study are listed as follows:

- Dual-branch signal processing framework: given the weak variations in rPPG signals, which are easily disturbed, we designed and implemented a dual-branch signal processing framework that combines static and dynamic features through an efficient feature fusion module, enhancing both robustness and overall processing efficiency.
- Long-range temporal feature extraction module: to capture the sequential characteristics in video sequences and increase temporal depth, we propose a single and double-frame alternate time-shift module (ATSM), which strengthens the capture and analysis of temporal information, significantly improving inference accuracy.
- Multiscale feature fusion: to address the issue of feature loss during inference, we proposed a multiscale feature fusion strategy that integrates features extracted at different scales, allowing the model to capture subtle variations in blood flow more accurately.

## 2. Related Work

### 2.1. Dual-Branch Video Inference Network Architecture

The dual-branch video inference network architecture is a deep learning framework that processes video data through two distinct pathways, focusing separately on temporal and spatial features. This design enables comprehensive video understanding and inference by integrating these features.

The concept of a dual-branch video inference network was first proposed in [31], which introduced a “slow-fast” architecture. The core idea is to capture different spatiotemporal scales of information through two network branches operating at different speeds, thereby achieving more thorough and efficient video understanding. The advantages of this approach include capturing spatiotemporal information at various granularities through branches operating at different speeds, enabling comprehensive video content understanding, and using lateral connections for feature fusion to enhance information complementarily at different scales. Dual-branch network structures are widely adopted for this purpose. For instance, ref. [32] reviewed various methods that employ dual-branch structures in visual language pretraining and analyzed how dual-branch architectures preserve the unique characteristics of image and text modalities. This paper also suggests further exploration of fusion mechanisms and alignment between modalities to improve performance in visual language understanding. In [33], a dual-mask strategy was proposed, applying masking and reconstruction simultaneously to better capture the spatiotemporal dependencies in video data. The experimental results demonstrated that dual-mask strategies significantly outperformed single-mask approaches in terms of model performance, showing stronger spatiotemporal representation capabilities. In [34], the concept of masked autoencoders was extended to spatiotemporal data learning by introducing a dual-branch encoder structure, which better models the dynamics and spatial structure of time-series data.

The dual-branch video inference network architecture has been extensively applied in many fields, including video classification, event detection, behavior analysis, video summarization and retrieval, autonomous driving, traffic analysis, and medical image analysis. In these application scenarios, the architecture excels at processing both short- and long-term information in videos, delivering precise and robust results in dynamic and complex environments. In recent years, several related studies have emerged in the field of rPPG. In [28], a dual-branch multitask learning framework was proposed, with one branch being used to extract spatial features of facial videos and the other to model the temporal dynamics of rPPG signals. Through feature fusion and interaction, this framework simultaneously estimates multiple physiological data types, including heart rate and respiration rate. In [35], a dual-branch generative adversarial network was proposed for remote physiological measurements, where one generator extracted a blood volume pulse (BVP) signal from facial videos, while the other extracted noise signals. By jointly optimizing both generators, the BVP signal and noise were better separated, thereby improving rPPG accuracy. Both studies adopted dual-branch structures to extract different features through two parallel branches, followed by fusion, achieving superior performance compared to single-branch networks.

### 2.2. Spatiotemporal Feature Fusion

Video streams contain spatial information (static features) in each frame and temporal information (dynamic features) between frames. Spatiotemporal feature fusion involves the effective combination of these two types of information to achieve more accurate and comprehensive video understanding. Common methods for spatiotemporal feature fusion include 3D CNN [36], ST-GCN [37], and TSM [38].

Recently, 3D CNNs have garnered significant attention in the rPPG field. In [26], 3D CNNs employ spatiotemporal convolutions to deal with facial features across both spatial and temporal dimensions, respectively, yielding good results in rPPG signal extraction and heart rate estimation tasks. However, 3D CNNs also have problems, including high computational complexity, large parameter sizes, and a limited ability to model long-term dependencies [39,40].

ST-GCN has also been increasingly applied in the rPPG field. In [41], Zitong Yu proposed a method of estimating heart rate with utilizing ST-GCN to analyze highly compressed facial videos. The rPPG signal inherently exhibits a graphical structure, such as the spatial topology and temporal evolution relationships between different facial regions. ST-GCN effectively models these spatiotemporal dependencies and adaptively extracts discriminative features from the rPPG signals. However, ST-GCN has limitations in handling facial motion and occlusion, and its generalization ability in complex environments requires further improvement [42,43].

To address the limitations mentioned in the previous two paragraphs, the study in [38] proposed the Temporal Shift Module (TSM), which introduces temporal information into spatial convolution operations through simple temporal shifts. This approach replaces temporal convolutions with index rearrangements, thereby avoiding multiple operations. As a result, the TSM achieves temporal feature extraction and fusion with relatively low computational overhead, enhancing the model’s temporal modeling ability while maintaining high efficiency. Considering factors such as computational efficiency, parameter size, transfer learning ability, and long-term dependency modeling, TSM emerges as a more efficient method for spatiotemporal fusion.

The specific operations are shown in Equations (1)–(3): for input feature map *X*, the operations are as follows:

(1) $$\begin{array}{c} X^{*}\left[:,:\alpha_{1}C,0:T-1,:,:\right]=X\left[:,:\alpha_{1}C,1:T,:,:\right] \end{array}$$

(2) $$\begin{array}{c} X^{*}\left[:,\alpha_{1}C:2\alpha_{1}C,1:T,:,:\right]=X\left[:,\alpha_{1}C:2\alpha_{1}C,0:T-1,:,:\right] \end{array}$$

(3) $$\begin{array}{c} X^{*}\left[:,2\alpha_{1}C:,:,:,:\right]=X\left[:,2\alpha_{1}C:,:,:,:\right] \end{array}$$

In the above equations, *α*1 denotes the proportion of feature shift, *X* refers to the input feature map, X* is the new tensor obtained from the shifted part, and *C* stands for the total channels number. Equation (1) moves the [0, *α*1 *C*) part of the original tensor backward by one frame, Equation (2) moves the [*α*1 *C*, 2*α*1 *C*) part of the original tensor forward by one frame, and Equation (3) keeps the remaining part unchanged. After the movement, the vacancy was filled with 0 by default.

Since its introduction, the TSM module has shown excellent results in video-understanding tasks, demonstrating strong temporal modeling capabilities. For instance, ref. [44] designed a discriminative temporal shift module, which enhances the ability of capturing complex temporal dynamics by incorporating a subtraction operation before the temporal shift. This modification significantly improves performance in action recognition tasks.

Some researchers have already started applying these advanced spatiotemporal fusion modules to rPPG signal processing and physiological parameter estimation. The study in [27] leveraged the advantages of TSM in spatiotemporal modeling, effectively improving rPPG signal feature extraction and physiological parameter estimation performance by introducing temporal attention mechanisms and multitask learning strategies, while also achieving efficient model deployment.

### 2.3. Multiscale Feature Fusion

Multiscale feature fusion is a crucial research direction in deep learning, aimed at leveraging different scales’ features to improve the performance of visual tasks.

Early methods primarily used image pyramids, such as Gaussian pyramids, to obtain multiscale images [45] and extracted and fused features at various scales. Although these methods are simple and intuitive, they lack learning capability, which limits the effectiveness of the fusion process. With developing deep learning, researchers began using CNNs to learn multiscale feature representations [46]. They achieved feature fusion by integrating multiscale structures into different network layers. A Fully Convolutional Network (FCN) [47] pioneered the end-to-end semantic segmentation paradigm, followed by numerous improved multiscale fusion modules. The study in [48] proposed a pyramid pooling module that performs global average pooling and upsampling at different scales on convolutional feature maps, generating multiple feature maps with varying receptive fields and concatenating them with the original feature map to achieve global and local feature fusion. In [49], a feature fusion module was designed, enabling the interaction and fusion of information from different hierarchical levels. To further enhance the capability of multiscale representation and fusion, researchers have explored dynamic and adaptive fusion mechanisms. In [50], multiscale regions were adaptively sampled based on input instances to avoid redundant computations, thereby improving efficiency and fusion effectiveness.

Multiscale feature fusion techniques are also widely used in rPPG, with related studies demonstrating that multiscale fusion enhances the effectiveness and adaptability of multiscale representations. The study in [37] proposed a 3D convolutional neural network that integrates multiscale features to better capture the spatiotemporal characteristics in videos, thereby improving the accuracy of extracting the rPPG signal. The study [51] discussed various signal processing methods in rPPG technology, including the application of multiscale feature fusion to enhance signal robustness and extraction accuracy.

## 3. Architecture

**Figure 1:**
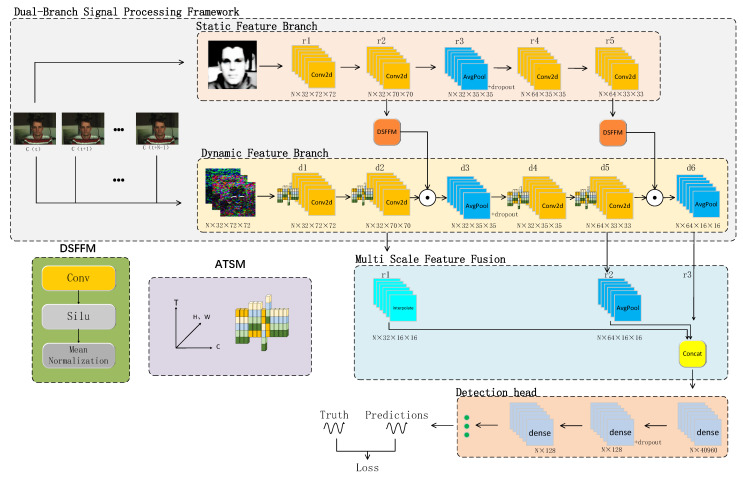
MDAR network structure diagram.

Figure 1 illustrates the structure of MDAR, which comprises two parallel processing channels: static and dynamic. These channels integrate information using a Dynamic Feature Integration Module (DSFFM). The static feature branch deals with spatial features, uses multi-level convolution (such as r1, r2, r3, r4, r5) to extract features at different levels, refines feature information layer by layer, and gradually captures the details and structure of the image from global to local to ensure the comprehensiveness and accuracy of feature extraction. The dynamic feature branch deals with time-series data, and four ATSM modules are introduced to enhance the temporal depth of features and balance the extraction accuracy of temporal features and computing resource consumption. In addition, multiscale feature fusion is applied in the dynamic feature branch after feature extraction, and downsampling is performed by interpolate and AvgPool to reduce the size of the feature map and extract higher level semantic information. Finally, the heart rate waveform is inferred through a dense layer, and the difference between the inferred waveform and the actual label serves as the loss function. The network parameters are iteratively optimized by minimizing this loss function. Dropout is used for regularization in the network to ensure the generalization ability.

By introducing standard deep learning architectures such as Conv2D, average pooling, dense, and dropout models can extract local features more efficiently and further reduce the data dimension, thereby enhancing the ability to capture key physiological signals in the input data. Next, we describe in detail the mathematical equations and specific roles of these key components. The specific operations are shown in Equations (4)–(7), respectively.

(4) $$O_{i,j}=\sum_{m=0}^{k}\sum_{n=0}^{k}l_{i+m,j+n}\cdot K_{m,n}+b$$

Equation (4) is the calculation formula of Conv2D, which extracts the local features in the image by sliding the convolution kernel on the image and performing multiplication and addition operations. In the equation, *O_i,j_* represents the value of row *i* and column *j* in the output two-dimensional data, *l* indicates the input two-dimensional data, *K* stands for convolution kernel, *b* means offset, and *k* is the size of the convolution kernel.

(5) $$y\left(i,j\right)=\frac{1}{k^{2}}\sum_{m=1}^{k}\sum_{n=1}^{k}x(i+m-1,j+n-1)$$

Equation (5) is a tie pooling formula, which reduces the dimension of the feature map by calculating the average value of the elements in the pooling window. In Equation (5), *y*(*i,j*) is the value of the output feature map at the position (*i*,*j*), *x*(*i* + *m* − 1, *j* + *n* − 1) is the pixel value of the input feature map in the pooled window, *k* is the size of the pooled window, and all pixel values in the pooled window are averaged to obtain the corresponding output value of the window [52].

(6) y=f(Wx+b)

Equation (6) is a dense formula whose main function is to map input vectors to output vectors. In Equation (6), *y* is the output vector representing the output of the current layer, *f* is the activation function for introducing nonlinearities, *b* is the bias vector, *x* is the input vector, and *W* is the weight matrix.

(7) y=f(pWx+b)

Equation (7) is the dropout regularization formula, a technique that prevents overfitting by randomly dropping a subset of neurons in a neural network. In Equation (7), *y* is the output, *f* is the activation function, *p* is the dropout mask, *W* is the weight matrix, *x* is the input, and *b* is the bias term [53].

### 3.1. Dual-Branch Signal Processing Framework

To fully understand the input rPPG signals, we designed and implemented a novel dual-branch signal-processing framework that addresses the issue of weak and easily disturbed signals. This framework effectively combines the extraction and analysis of static and dynamic features, enabling the neural network to better capture and utilize the diverse types of information embedded in video signals, thereby improving overall analytical accuracy and robustness.

The “Static Feature Branch”, labeled in Figure 1, extracts features from the input using a series of 2D convolutional layers (Conv2D) to capture the static appearance features in video frames. Average pooling layers (AvgPool) are inserted between the convolutional layers. This branch focuses on capturing the spatial details and texture information within individual frames, extracting stable, time-independent visual features that provide rich contextual information for subsequent processing steps, thereby enhancing the overall performance of the network.

The “Dynamic Feature Branch”, shown in Figure 1, extracts features through convolutional and average pooling layers. Before applying Conv2D, a custom ATSM module is introduced to perform temporal fusion of adjacent frame features and capture dynamic changes. This branch is responsible for analyzing temporal variations between consecutive frames and capturing small motion patterns and temporal information, making it particularly suitable for detecting subtle changes related to physiological signals.

During the feature extraction process, the dual-branch structure, with each branch having a distinct focus, captures different aspects of the data. We designed a dynamic–static feature fusion module (DSFFM) for fusing the outputs of two branches. As shown in Figure 1’s DSFFM, this module first applies a convolution (kernel size 1) to a tensor from static branch and then performs a nonlinear transformation using the SiLU activation function to enhance the expressive power of model. The resulting features are averaged to produce a one-dimensional value. Finally, the static and dynamic features are integrated by element-wise multiplication, as described in Equation (8):

(8) $$\begin{array}{c} X^{*}=r_{n}⨀\left(d_{n}\cdot \beta \right) \end{array}$$

where *r_n_* represents the features from the static branch, *d_n_* represents the features from the dynamic branch, *β* is a scaling factor for the static features, X* represents the fused output features, and ⨀ means the multiplication of corresponding elements of vectors *r_n_* and (*d_n_* × *β*). This scaling mechanism effectively balances the static and dynamic features, making the fused features better suited for downstream tasks.

In the overall framework, we fused both high-resolution features (from r2 in the static branch and d2 in the dynamic branch) and low-resolution features (from r5 in the static branch and d5 in the dynamic branch) using the DSFFM. This fusion strategy integrates static and dynamic features at both shallow and deep feature layers, retaining local details in the lower layers while also incorporating global semantics in the higher layers. This approach significantly enhances the richness and discriminative power of the feature representations.

### 3.2. ATSM

**Figure 2:**
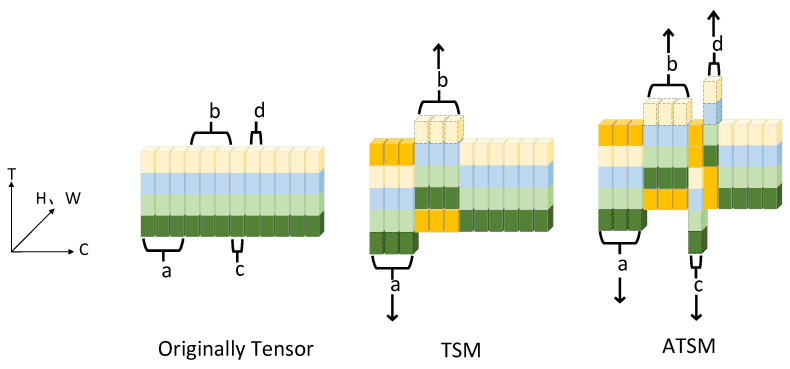
ATSM network structure diagram.

Because rPPG signals reflect blood flow changes during heartbeats, they exhibit stronger regularity and longer temporal dependencies than general video signals. The TSM module shifts the original tensor only once, primarily focusing on short-term dependencies between adjacent frames, which may result in missing finer temporal details of the rPPG signal and provides limited predictive power for long-term temporal trends. To address this issue, we propose an ATSM, as shown in Figure 2. This module introduces a two-frame shift in addition to the original one-frame shift, optimizing the architecture by incorporating multiple layers of temporal shifts and enabling the capture of longer-range temporal dependencies.

In Figure 2, C represents the channel dimensions. T is the temporal dimensions. H is the height of the feature map. W stands for the width. Unlike the TSM, which shifts some parts one frame backward and others one frame forward, the ATSM shifts part a one frame backward, part b one frame forward, part c two frames backward, and part d two frames forward, while the remaining parts remain unchanged. The dashed boxes in Figure 2 represent the discarded features, and the orange parts indicate zero padding. The key difference between ATSM and TSM lies in ATSM’s more flexible and diverse shifting mechanism. While TSM only shifts by one frame in either direction, ATSM shifts features by varying magnitudes (one or two frames), enabling it to capture deeper temporal dependencies and long-range dynamics.

The operation of ATSM is detailed in Equation (9):

(9) $$\begin{array}{c} X^{*}\left(n,c,h,w,t\right)=\left\{\begin{array}{l} \begin{array}{cc} X\left(n,c,h,w,t+1\right), & \;\;\;\;\;\;\;\;\;\;\;\;\;\;\;\;\;\;\;\;\;\;\;\;\;\;\;0\leq c<\alpha_{1}C\;\;\;\;\;\;\;\;\;\;\;\;\;\;\;\;\;\;\;\;\;\;\;\;\;\;\;\; \end{array}\\ \begin{array}{cc} X\left(n,c,h,w,t-1\right),\;\; & {\;\;\;\;\;\;\;\;\;\;\;\;\;\;\;\;\;\;\;\;\;\;\alpha }_{1}C\leq c<2\alpha_{1}C\;\;\;\;\;\;\;\;\;\;\;\;\;\;\;\;\;\;\;\;\;\;\;\;\;\; \end{array}\\ \begin{array}{cc} X\left(n,c,h,w,t+2\right), & \;\;\;\;\;\;\;\;\;\;\;\;\;\;\;\;\;\;\;2\;\alpha_{1}C\leq c<2\alpha_{1}C+\propto_{2}C\;\;\;\;\;\;\;\;\;\;\;\; \end{array}\\ \begin{array}{cc} X\left(n,c,h,w,t-2\right), & \;\;\;\;\;\;2\alpha_{1}C+\propto_{2}C\leq c<2\alpha_{1}C+{2\propto }_{2}C\;\;\;\;\;\;\; \end{array}\\ \begin{array}{cc} X\left(n,c,h,w,\;\;\;\;t\;\;\;\;\right), & \;2\alpha_{1}C+{2\propto }_{2}C\leq c\leq C\;\;\;\;\;\;\;\;\;\;\;\;\;\;\;\;\;\;\;\;\;\;\;\;\;\;\;\;\;\;\;\;\; \end{array} \end{array}\right. \end{array}$$

Here, *α*_1_ and *α*_2_ represent the proportions of the feature shift; *X* represents the input feature map. *n* is batch size. *c* indicates channel dimension. *t* denotes temporal dimension. h represents height. *w* is width. X* represents the new tensors obtained after the shift operation. In Equation (9), the first part of the tensor is shifted one frame backward, the second part is shifted one frame forward, the third part is shifted two frames backward, and the fourth part is shifted two frames forward, while the remaining part remains unchanged. All shift operations fill the resulting gaps with zero values to maintain the shape of the tensor.

By alternately applying single- and double-frame shifts, the ATSM captures dynamic changes in videos across different temporal scales. Single-frame shifts focus on capturing fine-grained changes between adjacent frames and detecting subtle and rapid movements in the videos. In contrast, double-frame shifts are designed to model more coarse-grained motion patterns over longer timeframes, capturing slower dynamic changes in the video. This multiscale temporal modeling approach comprehensively captures temporal dependencies in videos, enhances the model’s understanding of complex dynamic scenes, and significantly improves performance in complex temporal tasks.

### 3.3. Improved Multiscale Feature Fusion

Although the dual-branch structure incorporates feature fusion, subtle variations in rPPG signals may still lose some details as the network deepens. To address this issue, we design a multiscale fusion method tailored for rPPG signal processing. This method effectively fuses features at multiple scales across different network layers, fully leveraging the complementarity and synergy of information at various granularities. This multiscale feature fusion strategy not only preserves shallow detail features but also enhances deep semantic features, thereby improving the model’s ability to represent subtle signals in complex scenarios.

As shown in the “multiscale feature fusion” of Figure 1, we designed a multiscale fusion mechanism in the network by downsampling the global features from the shallowest layer d2 and intermediate layer d5, and then concatenating them with the deep features from d6. This fusion method integrates feature maps from different depths and receptive fields. Specifically, the shallow feature d2 has smaller receptive fields, capturing fine local variations in the video, while the deep feature d6 has the largest receptive fields, capturing broad global semantic information and temporal patterns. The intermediate features, d5, serve as a balance between the shallow and deep features, capturing mid-level patterns. By organically fusing these multiscale features, the model can more comprehensively describe the different frequency components and amplitude variations in rPPG signals, thereby improving signal extraction accuracy and robustness. This method not only enhances the model’s adaptability to complex dynamic scenes but also significantly improves its ability to detect subtle physiological changes, providing a solid foundation for high-precision rPPG signal analysis.

## 4. Experiments

### 4.1. Experimental Setup

We followed the official configuration of the rPPG toolbox for all the experimental parameter settings [54]:

- Batch size: 4
- Epochs: 30
- Initial learning rate: 0.01
- Weight decay: 9 × 10^−3^

The accuracy of the model can be evaluated by two metrics: mean absolute error (MAE) and mean absolute percentage error (MAPE). The equations for MAE and MAPE are shown in Equations (10) and (11), respectively.

(10) $$\begin{array}{c} MAE=\frac{1}{N}\sum_{i=1}^{N}\left|{\hat{y}}_{i}-y_{i}\right| \end{array}$$

(11) $$\begin{array}{c} MAPE=\frac{100\%}{N}\sum_{i=1}^{N}\left|\frac{{\hat{y}}_{i}-y_{i}}{y_{i}}\right| \end{array}$$

where *N* is the number of samples, $y_{i}$ is the true heart rate of the *i*-th sample, and ${\hat{y}}_{i}$ is the predicted heart rate of the *i*-th sample. Both MAE and *MAPE* are frequently used to measure the deviation between the predicted and true values. The reduction of *MAE* and *MAPE* indicates that the prediction ability of the model is enhanced.

### 4.2. Datasets

In our study, we selected three representative datasets: UBFC-rPPG [55], PURE [56], and MMPD [57], to evaluate the effectiveness and generalizability of our method. The PURE dataset (440 citations), UBFC-rPPG dataset (363 citations), and the recently published MMPD dataset (already 25 citations) are widely used benchmarks for evaluating the performance of rPPG methods.

PURE consists of 60 videos from ten participants (eight males and two females). The participants were asked to perform various head movements in front of the camera, and 60 one-minute sequences were recorded to capture head images and reference pulse measurements. The PURE dataset includes a variety of movement tasks, such as maintaining a stable state, talking, and performing head translation and rotation. This dataset provides a comprehensive and challenging platform for researchers, and typically serves as a test of the robustness of rPPG methods.

UBFC-rPPG contains 42 videos recorded at 640 × 480 pixels. Participants were required to play a heart rate-raising game to simulate human–computer interaction scenarios. This dataset is often regarded as a reliable benchmark for evaluating the performance of rPPG.

The MMPD dataset consists of 11 h of mobile phone recordings from 33 participants, annotated with eight descriptive labels: lighting, skin tone, exercise, movement, gender, eyeglasses, makeup, and wig. This dataset includes two subsets: a full dataset (240 × 320 resolution) and a mini-dataset (60 × 80 resolution). We used the full dataset to evaluate the algorithm’s performance more comprehensively.

We adopted a cross-dataset testing strategy [58] to further validate the generalization capability. We trained the network by one dataset (e.g., PURE) and tested on another (e.g., UBFC-rPPG). This cross-validation strategy allows for an effective network evaluation for unseen data, simulating potential data distribution differences encountered in real-world applications. In all experiments, the training set was split into two parts for training and validation (8:2 ratio), while the test set was used entirely for testing.

In this study, we treated the MMPD dataset as a pure test set and did not use it for training. The uniqueness of the MMPD dataset lies in its data collection under various scenarios and conditions. Smaller datasets are for training (PURE and UBFC-rPPG), and the larger for testing (MMPD). Hence, we were able to more comprehensively assess the robustness and generalization ability of the algorithm.

To further evaluate the model’s generalization capability, in addition to conducting hyperparameter experiments, all experiments presented in this study were validated using four distinct combinations of training and testing datasets: PURE is for the training and UBFC-rPPG for testing; UBFC-rPPG is for the training with PURE as testing; PURE is for the training and MMPD for testing; UBFC-rPPG is for the training with MMPD as testing.

**Figure 3:**
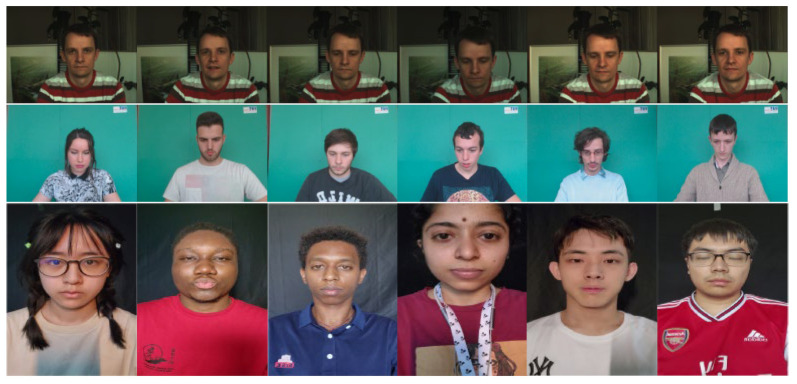
Sample dataset pictures.

Figure 3 shows example images from these datasets. These first line images in Figure 3 are from PURE, including participant’s various movements. The second row shows examples from UBFC-rPPG with images of participants engaged in a heart rate-raising game. The third row contains examples from MMPD, featuring various conditions such as lighting, skin tones, exercise, sex, eyeglasses, makeup, and wig states. For instance, in the first and second images of the third row, one participant is wearing eyeglasses while the other is not, and in the fourth to sixth images, the participants have different skin tones. The diversity of these datasets provides significant benefits for validating the robustness and generalizability of the proposed model.

### 4.3. Experiment Results

In this section, we comprehensively assess the performance and robustness of our method. In Section 4.3.1, hyperparameter experiments are conducted to find out the optimal configuration for the model. In Section 4.3.2, ablation experiments are presented to determine the contribution of each component. Finally, Section 4.3.3 gives the comparative experiments to verify the strength of our method over existing state-of-the-art techniques.

#### 4.3.1. Hyperparameter Experiment

In the hyperparameter experiments, the smallest dataset, UBFC-rPPG, was used for training, while the largest dataset, MMPD, was used for testing. Training on a smaller dataset reduces risk of overfitting, and testing on a larger dataset evaluates the network’s robustness in diverse scenarios, thereby validating its performance under resource-constrained conditions.

Table 1 presents the experiments’ results with divers scaling factors in the DSFFM module, where the β values in Experiments 1–5 were set to 0.1, 0.2, 0.5, 1, and 2, respectively. The results indicate that when *β* = 0.2, both *MAE* and *MAPE* reached their lowest values, 15.5 and 17.2, respectively. This suggests that the DSFFM module performs best when *β* = 0.2, and thus we used the scaling factor from Experiment 2 for DSFFM in our method. In this study, the optimal values are highlighted in bold. The suboptimal conditions are underlined. This convention is maintained throughout all subsequent tables.

Table 2 presents the experimental results for different frame-shift parameters in the TSM and ATSM. Experiment 1 used the original TSM shift parameters, while Experiments 2 and 3 used modified parameters. Experiments 4–6 employed different shift parameters for the proposed ATSM. The results show that Experiments 4–6 outperform Experiments 1–3 in both MAE and MAPE, confirming the feasibility of alternating single- and double-frame shifts. When *α*_1_ = 1/4 and *α*_2_ = 1/12, ATSM achieves the best performance with MAE and MAPE values of 13.87 and 15.33, respectively, significantly reducing the error and demonstrating improved accuracy. Therefore, we selected the hyperparameters from Experiment 6 for the ATSM.

#### 4.3.2. Ablation Experiment

The results of the ablation experiments are presented in Table 3. Experiment 1 served as the baseline, utilizing a dual-branch signal-processing framework with the original TSM module and without multiscale feature fusion. In Experiment 2, the TSM module was replaced with the proposed ATSM module. Experiment 3 added the multiscale feature fusion module to the baseline setup. Finally, Experiment 4 incorporated both the ATSM and the multiscale feature fusion module, representing the full MDAR method proposed in this study.

Experiment 2, compared to the baseline Experiment 1, achieved the second-best scores in three out of four different training and testing dataset comparisons, significantly enhancing performance. Experiment 3 showed a slight improvement over Experiment 1, particularly when PURE was used for the training set and UBFC-rPPG for testing, achieving the second-best scores. Experiment 4, which represents the final method, exhibited the lowest *MAE* and *MAPE*, outperforming all other combinations. These results indicate substantial improvements in predictive capabilities and increased robustness through incremental experimental optimization. Notably, while multiscale feature fusion alone resulted in modest performance gains, its combination with ATSM yielded significant improvements, a phenomenon commonly observed in deep learning [59].

The ablation experiments confirmed that every improvement of MDAR significantly contributes to performance. The ATSM module enhances temporal feature extraction, while the multiscale feature fusion module improves representations of spatial and temporal features, resulting in more accurate predictions of heart rate.

#### 4.3.3. Comparison Experiment

In this section, we conducted comparison experiments with currently main rPPG methods, including DeepPhys [16], EfficientPhys [17], PhysFormer [18], PhysNet [19], and TS-CAN [20]. Table 4 presents the results.

By comparing the performances of different methods across four combinations of training and testing datasets, the MDAR method achieved three first-place and one second-place results, demonstrating a clear overall advantage with robust performance and strong generalization capability. While the PhysNet algorithm performed best when using UBFC-rPPG for training and MMPD for testing, its performance was poor for the other three combinations. Additionally, the inference speed of MDAR is 0.5 ms per frame, which is comparable to TS-CAN and DeepPhys, meeting the real-time requirement and outperforming PhysFormer and EfficientPhys.

**Figure 4:**
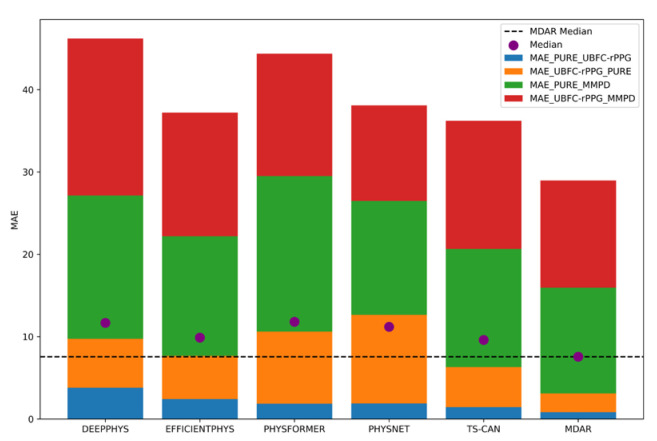
MAE stack bar plot.

**Figure 5:**
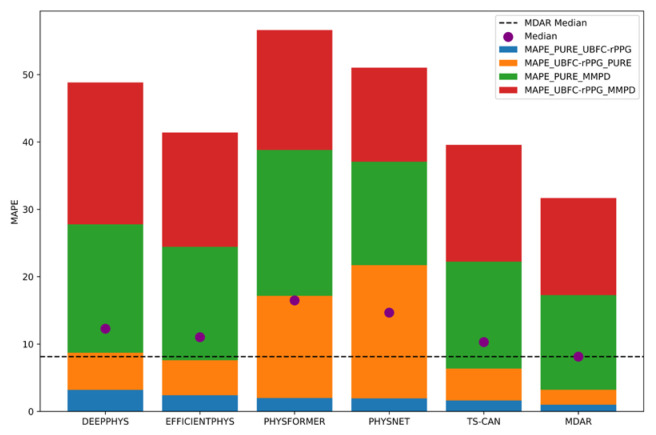
MAPE stack bar plot.

To provide a more intuitive comparison of results to other advanced methods, we plotted the comparison as stack bar plots, as shown in Figure 4 and Figure 5.

Figure 4 and Figure 5, respectively, illustrate the *MAE* and *MAPE* values across different methods in four cross-dataset experiments. The different colors in the figure represent the *MAE* and *MAPE* of the model on different training sets and testing sets, respectively; for example, the blue rectangle in the legend box within the upper right corner of Figure 4 indicates that the first item (PURE) is used as the training set, and the second item (UBFC-rPPG) is used as the testing set. The different color sections of the MDAR bar are, respectively, smaller than most other models in Figure 4 and Figure 5 (except that PhysNet slightly outperforms MDAR in the red section), especially on the blue section (PURE_UBFC-rPPG) and green section (PURE_MMPD) cross-dataset experiments. Additionally, the overall height of the MDAR bars is the lowest, indicating that, when considering all four cross-dataset results, MDAR performs optimally. This shows that MDAR performs better on various test sets, showing strong robustness. In addition, the median of MDAR is also lower than the median of other models, which is easily seen by the dotted line. This is further evidence that MDAR’s overall performance is excellent. A comprehensive analysis shows that the MDAR method achieved the lowest *MAE* and *MAPE* among all methods, making it the most accurate algorithm overall.

To comprehensively and systematically evaluate the advantages of MDAR over other methods, we computed the performance improvement percentage and average improvement value for each method. For each experiment, we calculated the percentage improvements in *MAE* and *MAPE* for MDAR relative to the other methods, denoted as MAEp and MAPEp, respectively. This approach provides a clear visualization of MDAR’s superiority across different experimental scenarios. We also calculated the average improvement percentages for $\bar{MAEp}$ (the average percentage improvement in *MAE*) and $\bar{MAPEp}$ (the average percentage improvement in *MAPE*) across all methods. These two metrics reflect MDAR’s average advantage in reducing errors.

As shown in Table 5, the MDAR demonstrates a clear advantage over other methods in cross-dataset experiments, with an average improvement in *MAE* ranging from 30.6% to 49.4%, and an average improvement in MAPE ranging from 30.2% to 47.4%. Compared with TS-CAN, EfficientPhys, DeepPhys, and PhysFormer, all metrics showed significant improvements.

The analysis from multiple perspectives clearly demonstrates that the proposed MDAR outperforms currently advanced approaches in accuracy and robustness. This comprehensive evaluation further validates MDAR’s superior performance, highlighting its ability to identify long-range temporal relationships and fuse features across different scales. These capabilities make MDAR more robust and precise than existing methods, particularly in complex environments and diverse datasets.

**Figure 6:**
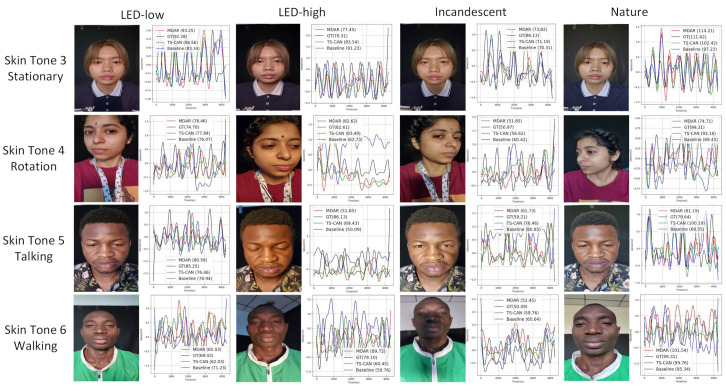
Visual example diagram.

Figure 6 presents a series of visual examples under varying conditions, such as different lighting, skin tones, and motion conditions. These examples compare the predicted heart rate values generated by three methods—MDAR, TS-CAN, and the baseline architecture used in ablation Experiment 1—with the ground truth (GT) heart rate. TS-CAN, selected for comparison due to its superior overall performance among other algorithms, is juxtaposed with MDAR. Consistent with the setup of the hyperparameter experiment, all examples were selected from the MMPD dataset, and the model inference was based on the UBFC-rPPG dataset, which was used as the training set.

At the top of Figure 6, different lighting conditions are indicated, while the left side includes various skin tones and motion states. The predicted heart rates for each algorithm are shown in parentheses next to the algorithm names. For example, in the first row, first example, the actual heart rate is 92.28, while the predicted heart rates from MDAR, TS-CAN, and the baseline are 93.25, 98.56, and 83.34, respectively. As demonstrated, the MDAR method outperforms TS-CAN and the benchmark method in most scenarios. This superior performance is particularly evident in the first row (first and fourth examples), the second row (second and third examples), and the third row (third and fourth example), as well as in the fourth row (first and second examples).

Although MDAR has relatively weaker inference results in some cases, such as in the second row, fourth example, where TS-CAN performs better, and in the fourth row, first example, where the baseline method outperforms MDAR, it still demonstrates more stable performance overall. MDAR generally provides more consistent and reliable heart rate predictions across various conditions (different lighting, skin tones, and motion conditions) compared to the other methods.

## 5. Discussion

In this study, MDAR significantly enhances the accuracy and robustness of the remote estimation of human heart rate while maintaining real-time inference speed. Through cross-dataset validation, it was found that MDAR consistently outperforms existing approaches in terms of *MAE* and *MAPE* on three public datasets: UBFC-rPPG, PURE, and MMPD. The PURE dataset includes a variety of movement tasks, UBFC-rPPG covers scenarios with a wider range of heart rates, and MMPD provides a diverse set of comparison samples, specifically highlighting differences in lighting, skin tone, exercise, movement, and gender, as well as the presence of eyeglasses, makeup, and wigs. These benchmark datasets are designed to better reflect the performance of algorithms in real-world, complex environments.

In particular, the prediction results of MDAR on these datasets are closer to the actual true heart rate, leading to improved performance in both *MAE* and *MAPE* metrics. Specifically, compared to state-of-the-art methods, MDAR achieves average improvements in *MAE* and *MAPE* of 30.6–49.4% and 30.2–47.4%, respectively, demonstrating strong generalizability across various experimental scenarios for remote heart rate estimation. Moreover, MDAR maintains good real-time performance while ensuring high accuracy, making it suitable for application scenarios that require fast response times.

The performance improvements are mainly attributed to the in-depth analysis of the rPPG data and the targeted architectural design. MDAR is built on a dual-branch architecture that leverages the separation of static and dynamic features, thereby improving the model’s ability to extract and cope with different types of data. The ATSM module leverages the characteristics of long-term temporal dependencies and proposes an innovative approach that incorporates a small portion of two-frame shifts into the traditional single-frame shift. This design effectively extends the model’s capacity to capture long-term dependencies, enabling more accurate extraction of temporal features when detecting subtle physiological signals such as heart rate. Compared to other models that rely on adjacent frame information, the ATSM enhances the network’s ability to perceive temporal dependencies, significantly improving the ability to handle varying environmental conditions and lighting. Inspired by our previous research on feature fusion, multiscale feature fusion was applied to a dual-branch architecture for the first time [60]. Multiscale feature fusion integrates features across different network layers, allowing the network to understand local and global semantic features, which provides a more robust feature representation for extracting rPPG signals. These design elements complemented each other, significantly enhancing the overall performance and adaptability of the algorithms, thereby improving the model’s generalization across different skin tones, lighting conditions, and scene variations.

Compared with single-branch algorithms such as DeepPhys, PhysNet, and PhysFormer, which rely on a single feature channel, these methods struggle to fully capture the interaction between static facial textures and dynamic blood flow changes in rPPG signals. In contrast, MDAR uses a dual-branch architecture to separately process static features (such as facial color and texture) and dynamic features (such as blood flow changes), significantly enhancing the model’s signal extraction accuracy and adaptability to complex scenarios, while maintaining a relatively low computational cost.

Compared to dual-branch algorithms such as TS-CAN, MDAR offers more pronounced advantages in temporal feature extraction and multiscale feature fusion. MDAR employs an innovative ATSM module that combines single-frame and two-frame time shifts, further enhancing the model’s ability to capture temporal features. Moreover, the multiscale feature fusion strategy in MDAR allows the model to integrate information across different layers, making it more robust and generalizable across diverse datasets and complex environments.

Certainly, the performance improvements come with a slight increase in inference time. MDAR has an inference time of 0.5 ms, which is indeed slower than DEEPHYS at 0.33 ms and TS-CAN at 0.43 ms, due to the relatively larger model size than DEEPHYS and TS-CAN. Despite its slightly increased computational complexity, MDAR’s real-time capabilities remain within an acceptable range with an improvement of over 30% in both *MAE* and *MAPE* metrics. Therefore, even with a minor sacrifice in speed, MDAR’s efficient performance ensures its applicability in the field of high-precision heart rate monitoring.

A potential direction for the future development of this field is the integration of multimodal data alongside video signals for remote heart rate measurement. Current approaches primarily rely on single-modal data, which may not perform well in more complex conditions. For instance, in scenarios with harsher lighting, greater facial occlusions, or rapid movements, the robustness of the algorithm can significantly decrease. The limitations of single-modal approaches are evident in their sensitivity to external interference, leading to increased noise and unstable measurement results, thus limiting their potential in real-world applications. By incorporating additional physiological signals like respiratory rate, skin temperature, and motion data, the model’s generalization and accuracy could be significantly enhanced. Multimodal fusion could help reduce noise, improve signal reliability, and provide a more comprehensive solution for contactless physiological measurements, making the model more adaptable and stable in real-world scenarios.

Although the three datasets (UBFC-rPPG, PURE, and MMPD) come from real-world scenarios, our team could not verify the MDAR algorithm in the actual work scenario due to the lack of authorization from local hospitals. This limitation impacts our overall assessment of the model’s adaptability and robustness in real-world environments and restricts its potential value in various applications. We look forward to future studies with more real-world data to verify the validity of the model in practical applications. In collaboration with the medical industry, the stability and generalization ability of the model are evaluated more comprehensively through a combination of field experiments and long-term follow-up analysis.

In summary, the MDAR method demonstrates great potential for wide-ranging applications in remote heart rate measurement. It can be used not only for health monitoring but also for the unobtrusive monitoring of physiological signals from multiple individuals, making it particularly suitable for densely populated public places, security monitoring, and isolation wards. Furthermore, with advancements in sensor technology, MDAR is expected to be applied more broadly in fields such as healthcare, sports monitoring, and smart devices, providing efficient and accurate solutions for contactless health monitoring.

## 6. Conclusions

This study proposes the MDAR framework, a robust advancement in non-contact heart rate monitoring. Our approach significantly enhances the precision of remote photoplethysmography (rPPG) through innovative dual-branch architecture, advanced temporal feature extraction, and multiscale feature fusion. The dual-branch signal processing framework integrates both static and dynamic features, reducing the uncertainty in prediction results caused by the weak and easily disrupted nature of the signals. The alternate time-shift module (ATSM) captures long-range, deep-level variation patterns, effectively addressing the challenges posed by significant differences in skin absorption and reflection characteristics across different wavelengths. Additionally, the multiscale feature fusion method combines features at various granular levels, enhancing the robustness of the algorithm. Validated across three public datasets—UBFC-rPPG, PURE, and MMPD—MDAR achieves considerable improvements over existing models, confirming its efficacy and potential for broader application in complex environments.

MDAR shows great promise, yet its potential in real-world settings has not been fully realized due to the lack of local hospital authorization and the need for more advanced multimodal integration. Future work will benefit from exploring these avenues, particularly focusing on enhancing data robustness through the incorporation of diverse physiological and environmental signals, as well as fostering collaboration with hospitals.

Ultimately, our research paves the way for more accurate and adaptable rPPG technologies, setting the groundwork for their integration into everyday health monitoring and clinical diagnostics. As we continue to refine and test these models, the goal is to extend their reliability and applicability, ensuring they meet the rigorous demands of real-world medical and healthcare applications.

## Figures and Tables

**Table 1:** Results of *β* hyperparameter tuning experiments in DSFFM.

Experiment	*β*	MAE	MAPE
1	0.1	15.76	17.49
2	0.2	**15.5**	**17.2**
3	0.5	15.83	17.57
4	1	15.68	17.3
5	2	15.63	17.28

Optimal values are marked in bold, suboptimal values are marked in underline.

**Table 2:** Results of α_1_ and α_2_ hyperparameter tuning experiments in TSM and ATSM.

Experiment	Method	*α*1	*α*2	MAE	MAPE
1	TSM	1/8	N/A	15.66	17.24
2	TSM	1/4	N/A	16.00	17.67
3	TSM	1/3	N/A	16.64	18.54
4	ATSM	1/6	1/6	15.06	16.5
5	ATSM	1/8	1/12	15.52	16.96
6	ATSM	1/4	1/12	**13.87**	**15.33**

Optimal values are marked in bold, suboptimal values are marked in underline.

**Table 3:** Ablation experiment results.

Experiment	Training Set	Testing Set					
		PURE		UBFC-rPPG		MMPD	
		MAE	MAPE	MAE	MAPE	MAE	MAPE
1	PURE	N/A	N/A	3.24	3.22	14.37	15.63
	UBFC-rPPG	5.22	5.15	N/A	N/A	15.5	17.2
2	PURE	N/A	N/A	1.40	1.50	13.34	14.64
	UBFC-rPPG	4.84	4.77	N/A	N/A	13.87	15.33
3	PURE	N/A	N/A	0.98	1.11	14.33	15.58
	UBFC-rPPG	5.16	5.83	N/A	N/A	15.32	17.08
4	PURE	N/A	N/A	**0.83**	**0.99**	**12.86**	**14.05**
	UBFC-rPPG	**2.27**	**2.22**	N/A	N/A	**13.01**	**14.42**

Optimal values are marked in bold, suboptimal values are marked in underline.

**Table 4:** Comparative experimental results.

Method	Training Set	Testing Set						
		PURE		UBFC-rPPG		MMPD		Inference Time(ms)
		MAE	MAPE	MAE	MAPE	MAE	MAPE	
DeepPhys	PURE	N/A	N/A	3.8	3.19	17.41	19.05	**0.33**
	UBFC-rPPG	5.94	5.52	N/A	N/A	19.06	21.08	
EfficientPhys	PURE	N/A	N/A	2.42	2.39	14.52	16.84	2.73
	UBFC-rPPG	5.25	5.20	N/A	N/A	15.03	16.97	
PhysFormer	PURE	N/A	N/A	1.86	1.99	18.90	21.66	0.63
	UBFC-rPPG	8.75	15.17	N/A	N/A	14.86	17.79	
PhysNet	PURE	N/A	N/A	1.89	1.93	13.82	15.35	0.6
	UBFC-rPPG	10.77	19.78	N/A	N/A	**11.61**	**13.98**	
TS-CAN	PURE	N/A	N/A	1.44	1.63	14.34	15.86	0.43
	UBFC-rPPG	4.87	4.74	N/A	N/A	15.57	17.34	
MDAR	PURE	N/A	N/A	**0.83**	**0.99**	**12.86**	**14.05**	0.5
	UBFC-rPPG	**2.27**	**2.22**	N/A	N/A	13.01	14.42	

Optimal values are marked in bold, suboptimal values are marked in underline.

**Table 5:** Comparison of experimental growth percentage results.

Method	Training Set	Testing Set						$\bar{MAEp}$	$\bar{MAPEp}$
		PURE		UBFC-rPPG		MMPD			
		MAEp	MAPEp	MAEp	MAPEp	MAEp	MAPEp		
DeepPhys	PURE	N/A	N/A	78.1%	68.9%	26.1%	26.2%	49.4%	46.6%
	UBFC-rPPG	61.7%	59.7%	N/A	N/A	31.7%	31.6%		
EfficientPhys	PURE	N/A	N/A	65.7%	58.6%	11.4%	16.6%	36.8%	36.9%
	UBFC-rPPG	56.7%	57.3%	N/A	N/A	13.4%	15.1%		
PhysFormer	PURE	N/A	N/A	55.4%	50.1%	31.9%	35.1%	43.5%	47.4%
	UBFC-rPPG	74.1%	85.4%	N/A	N/A	12.4%	18.9%		
PhysNet	PURE	N/A	N/A	56.1%	48.7%	6.9%	8.5%	31.5%	35.7%
	UBFC-rPPG	78.9%	88.8%	N/A	N/A	−15.9%	−3.1%		
TS-CAN	PURE	N/A	N/A	42.3%	39.2%	10.3%	11.4%	30.6%	30.2%
	UBFC-rPPG	53.3%	53.2%	N/A	N/A	16.4%	16.8%		

## Data Availability

All data are available from the corresponding author upon reasonable request. The codes are available at https://github.com/crf0409/rPPG-MDAR (accessed on 20 October 2024).
